# Regular and low-dose aspirin, other non-steroidal anti-inflammatory medications and prospective risk of HER2-defined breast cancer: the California Teachers Study

**DOI:** 10.1186/s13058-017-0840-7

**Published:** 2017-05-01

**Authors:** Christina A. Clarke, Alison J. Canchola, Lisa M. Moy, Susan L. Neuhausen, Nadia T. Chung, James V. Lacey, Leslie Bernstein

**Affiliations:** 1 Cancer Prevention Institute of California, 2201 Walnut Ave. Suite 300, Fremont, CA 94538 USA; 2 Department of Health Research and Policy and the Stanford Cancer Institute, Stanford University School of Medicine, 150 Governor’s Lane, HRP Redwood Bldg, Stanford, CA 94305 USA; 3 Department of Population Sciences, Beckman Research Institute of City of Hope, 1500 East Duarte Rd, Duarte, CA 91010 USA; 4 Division of Research, Kaiser Permanente, 2000 Broadway, Oakland, CA 94612 USA

**Keywords:** Aspirin, NSAIDs, Breast cancer, Hormone receptor, HER2, Subtype, Epidemiology

## Abstract

**Background:**

Regular users of aspirin may have reduced risk of breast cancer. Few studies have addressed whether risk reduction pertains to specific breast cancer subtypes defined jointly by hormone receptor (estrogen and progesterone receptor) and human epidermal growth factor receptor 2 (HER2) expression. This study assessed the prospective risk of breast cancer (overall and by subtype) according to use of aspirin and other non-steroidal anti-inflammatory medications (NSAIDs) in a cohort of female public school professionals in California.

**Methods:**

In 1995 − 1996, participants in the California Teachers Study completed a baseline questionnaire on family history of cancer and other conditions, use of NSAIDs, menstrual and reproductive history, self-reported weight and height, living environment, diet, alcohol use, and physical activity. In 2005–2006, 57,164 participants provided some updated information, including use of NSAIDs and 1457 of these participants developed invasive breast cancer before January 2013. Multivariable Cox proportional hazards regression models provided hazard rate ratios (HRR) for the association between NSAID use and risk of invasive breast cancer as well as hormone receptor- and HER2-defined subtypes.

**Results:**

Developing breast cancer was associated inversely with taking three or more tablets of low-dose aspirin per week (23% of participants). Among women reporting this exposure, the HRR was 0.84 (95% confidence interval (CI) 0.72–0.98) compared to those not taking NSAIDs and this was particularly evident in women with the hormone receptor-positive/HER2-negative subtype (HRR = 0.80, 95% CI 0.66–0.96). Use of three or more tablets of “other” NSAIDs was marginally associated with lower risk of breast cancer (HRR = 0.79, 95% CI 0.62–1.00). Other associations with NSAIDs were generally null.

**Conclusion:**

Our observation of reduced risk of breast cancer, among participants who took three or more tablets of low-dose aspirin weekly, is consistent with other reports looking at aspirin without differentiation by dose. This is the first report to suggest that the reduction in risk occurs for low-dose aspirin and not for regular-dose aspirin and only among women with the hormone receptor-positive/HER2-negative subtype. This preliminary study builds on previous knowledge and further supports the need for formal cancer chemoprevention studies of low-dose aspirin.

## Background

Daily use of low-dose (81 mg) aspirin is formally recommended by the United States Preventive Services Task Force (USPSTF) for broad chemoprevention of cardiovascular disease [1] and colorectal cancer [2]. Regular-dose aspirin may also provide effective chemoprevention of breast cancer in women, although the evidence is mixed. In one clinical trial in which women were randomized to receive 100 mg of aspirin or placebo every other day, no reduction in breast cancer risk was observed among women receiving aspirin [3]. However, a meta-analysis published in 2008, which included 38 studies and over 2 million women, concluded that breast cancer risk overall was reduced in association with use of any aspirin or other non-steroidal anti-inflammatory drugs (NSAIDs) [4]. In contrast, in a more recent assessment of 32 studies the authors concluded that aspirin use was not associated with risk of breast cancer, although a statistically significant reduction in the risk of hormone receptor (HR)-positive subtypes was noted [5]. Both of these meta-analyses detected substantial heterogeneity of results among studies [4, 5]. One contributor to this heterogeneity may be variation in associations by breast cancer subtype, defined jointly by HR and HER-2/neu receptor (HER2) status. Tumor expression of these markers strongly influences clinical care (e.g., treatment with tamoxifen or trastuzumab) and is also associated with marked differences in incidence patterns and risk factors [6–9]. It is possible that NSAIDs differentially influence the development of tumors based on the expression of HR and HER2. Heterogeneity in the results for aspirin may also be explained by previous studies not distinguishing between low-dose or daily aspirin use, which are common patterns of NSAID use that may be misclassified in broader categorizations, such as all doses of ﻿aspirin combined or use of three or more tablets per week.

To date, one prospective and two case-control studies have examined detailed use of NSAIDs and risk of breast cancer subtypes defined jointly by HR and HER2 status. The Nurses’ Health Study reported that use of two or more tablets of any dose of aspirin per week was statistically significantly associated with the risk of the luminal A (HR-positive/HER2-negative) subtype but not associated with the luminal B subtype (HR-positive/HER2-positive), indicating the importance of categorizing HER2 in detail [10]. In the two case-control studies any aspirin use was statistically significantly associated with reductions in risk of all four breast cancer subtypes studied [11, 12] and one of these studies further suggested that reductions in risk were limited to overweight women [12], in whom adipose-related inflammation might be higher.

To add to the evidence on low-dose aspirin, other NSAIDs including regular dose aspirin (defined as 325 mg), and the risk of HR and HER2-defined breast cancer subtypes, we looked to our long-term prospective cohort of California public school professionals who were asked in 2005–2006 about current use of pain-relieving medications, including low-dose aspirin, regular-dose aspirin, ibuprofen, and other NSAIDs. A prior analysis of NSAIDS and breast cancer in this cohort [13] was based on earlier questionnaire data that did not differentiate between low-dose and regular-dose aspirin. With over 7 years of follow up of incident breast cancer (*n* = 1457) including crucial detail on tumor HER2 status since submission of the 2005–2006 questionnaire, we evaluated whether risk of breast cancer varied by recent use of NSAIDs, and explored whether any associations between NSAIDs and risk of breast cancer were modified by HER2-defined breast cancer subtypes or overweight status.

## Methods

The California Teachers Study cohort (CTS) was established in 1995–1996 when 133,479 active and retired female teachers, administrators and other public school professionals were recruited through the California State Teachers Retirement System [14]. Participants completed a baseline questionnaire that collected information on family history of cancer and other conditions, menstrual and reproductive history, self-reported weight and height, living environment, diet, alcohol and tobacco use, physical activity history, and frequency and duration of prior use of certain medications including aspirin (but without detail on aspirin dose).

In 2005–2006, a 10-year follow-up questionnaire collected updated information on frequency of current use of aspirin, low-dose aspirin and other pain-relieving medications (see below), weight, alcohol use, menopausal status, use of hormone therapy (HT), and physical activity. Copies of questionnaires are available at https://www.calteachersstudy.com/past-questionnaires. The CTS is overseen by the Institutional Review Boards of the Cancer Prevention Institute of California, the California Health and Human Services Agency, the University of California, Irvine, the University of Southern California, and the City of Hope.

CTS participants are followed annually for changes of address, cancer diagnoses, hospitalizations, outpatient surgeries, emergency room visits, and death. Annual linkage with the California Cancer Registry (CCR) is used to identify incident cancer among cohort members. The CCR is a population-based cancer registry, which is anchored in state legislation that mandates reporting and is estimated to be over 99% complete [15]. Annual linkages with the Office of Statewide Health Planning and Development (OSHPD) allow us to identify details of each members’ hospitalizations, outpatient surgeries, and emergency room visits. California and national mortality files are used to ascertain dates and causes of death.

### Breast cancer ascertainment

Information on all incident breast cancers was obtained from the CCR, including pathologic and clinical features, which are abstracted directly from the medical record. HR status was based on estrogen receptor (ER) and progesterone receptor (PR) status as routinely reported on diagnostic pathology records. HER2 status was also based on pathology report review. Subtypes were defined as follows: HR-positive/HER2-negative was defined as ER-positive or PR-positive and HER2-negative; HR-positive/HER2-positive was defined as ER-positive or PR-positive and HER2-positive; HR-negative/HER2-positive was defined as ER-negative and PR-negative and HER2-positive; and triple-negative was defined as ER-negative, PR-negative, and HER2-negative.

### Assessment of pain-relieving medications

On the 10-year follow-up questionnaire, women were asked whether they were currently taking any pain-relieving medications at least once a week, and if yes, the total number of tablets taken per week (1–2, 3–4, 5–6, 7–8, 9–10, 11–12, 13–14, 15–21, 22–28, or 29+ tablets per week). The medication choices included low-dose aspirin; aspirin or aspirin-containing product (Bayer, Bufferin, Excedrin); ibuprofen (Advil, Motrin); naproxen, ketoprofen or other non-steroidal (Aleve, Feldene, Indocin, Naprosyn, Orudis, Relafen); Cox-2 inhibitors (Celebrex, Vioxx); and acetaminophen (aspirin-free Excedrin, Tylenol, Tempra). Women were then asked if they had stopped regular use of any of these medications during the past 3 years, and if yes, why (by marking any of the following response categories that applied: “Condition improved", "Didn’t work", “I had side effects", “I heard about side effects", "Drug no longer available", “Other”). The baseline questionnaire asked if aspirin (Anacin, Bufferin, Excedrin) and ibuprofen (Advil, Motrin, Nuprin) were taken regularly (at least once a week), total years taken and how many days per week taken (1-3 days/week, 4-6 days/week or daily).

Each medication type from the 10-year questionnaire was initially categorized as “Never in the past 3 years", “Former", “Current" ,or “Unknown” for that type of medication. Women were classified into the category “Never in the past 3 years” if they reported: (1) current use of 0 or <1 medication per week or left this question blank; (2) never took the medication regularly or did not stop regular use or left this question blank; and (3) did not give a reason for stopping. “Current” users were those who reported: (1) current use of ≥1 tablet(s) per week; (2) “Never took regularly or did not stop use” or left this question blank; and (3) did not give a reason for stopping. “Former” users were those who reported: (1) “Yes, I stopped regular use” or gave a reason for stopping; and (2) reported current use as 0 or <1 medication per week or left this question blank. All other women, including those whose answers were inconsistent or who left both questions blank for all medications, were classified as “Unknown”.

For analysis, we: (1) focused on the five NSAIDs from the 10-year questionnaire, excluding acetaminophen; (2) grouped the women who were in the category of “Never in the past 3 years” for the medication of interest into women who reported (a) “No NSAIDs in the past 3 years,” which was used as the reference group, (b) no use of that particular type of medication but had used one or more of the other four NSAIDs in the past 3 years, and (c) no use of that particular type of medication and unknown use of one or more of the other four NSAIDS, which was combined with the “Unknown” category; and (3) combined former and current users of 1–2 tablets per week into one category. For low-dose aspirin, we also examined “Daily” use, which was defined as those with current use of 7+ tablets per week (Table 2).

### Study population

For the present analyses, we excluded women sequentially who at baseline were not residing in California (*n* = 8867), had a prior history of breast cancer (*n* = 6216), or had unknown cancer history (*n* = 135). We also excluded women who prior to the 10-year follow-up questionnaire had died (*n* = 8654), had requested no further contact from the CTS (n = 926), had moved out of California for more than 4 months (*n* = 8296), had developed breast cancer (*n* = 4188), or had a bilateral mastectomy without a diagnosis of breast cancer (identified from hospital discharge data) (*n* = 18). Among the remaining 96,179 participants, 57,164 (59%) completed the 10-year follow-up questionnaire. During follow up (from the date a woman completed the 2005–2006 questionnaire and continuing through 31 December 2012), 1457 women were diagnosed with invasive breast cancer after completing the 10-year follow-up questionnaire.

### Data analysis

Follow-up time was calculated as the number of days between the date the 10-year follow-up questionnaire was completed and the first of the following events: a first diagnosis of breast cancer (International Classification of Diseases for Oncology-3 (ICD-O-3) site code C50) (*n* = 1457 with invasive cancer; *n* = 393 in situ cancer), death (*n* = 3538), a move (for >4 months) out of California (n = 2082), bilateral mastectomy (*n* = 5), or 31 December 2012 (n = 49,689).

Hazard rate ratios (HRR) and 95% CI were estimated using multivariable Cox proportional hazards regression models, with age (in days) as the time metric and stratification by age (in years) at the time of the 10-year follow-up questionnaire. Covariates were included based on their independent association with risk for a given outcome with a *p* value <0.05 in multivariable models.

Competing risk analysis was used to estimate risk of invasive breast cancer overall and by different receptor subtypes. The 157 women diagnosed with breast cancer during follow up who had missing information on HR or HER2 status were excluded from all models with breast cancer subtype as the outcome. We tested the proportional hazards assumption for each covariate in the model and for the main effect for the different outcomes using a likelihood ratio test of interaction with the time metric (continuous age) based on cross-product terms. We found only one violation of the proportional hazards assumption: alcohol consumption had a statistically significant interaction with time-dependent age for HR-negative/HER2-negative tumors; thus, this interaction was included in the model for that outcome.

We conducted multivariable analyses in which we assessed the impact of adjusting for history of hospitalization for myocardial infarction (between 1991 and the date the 10-year follow-up questionnaire was completed based on ICD-9 diagnostic codes 410.00-410.92 from OSHPD linkage; no, yes) and history of diabetes mellitus (from the 10-year follow-up questionnaire; no, yes, missing). These adjustments did not meaningfully change the HRR when evaluating the association of current use of low-dose aspirin with risk of breast cancer overall or with risk of any of the receptor subtypes. Hence, history of myocardial infarction and history of diabetes mellitus were not included in the final models presented here. We also examined models of low-dose aspirin stratified by body mass index (BMI) (<25 or ≥25 kg/m^2^) and tested the interaction using a likelihood ratio test and cross-product terms, excluding women with unknown NSAID use.

In a secondary analysis, we considered aspirin use at baseline (regular-dose and low-dose aspirin were not asked about separately) in conjunction with use of regular-dose and low-dose aspirin from the 10-year questionnaire, with no NSAID use reported at both baseline (including aspirin and ibuprofen) and the 10-year follow-up (including regular-dose and low-dose aspirin, ibuprofen, Cox-2 inhibitors, and other NSAIDs) as the reference group. This analysis was limited to outcomes of breast cancer and the HR-positive/HER2-negative subtype, as we had an insufficient number of diagnoses to examine these associations in the other subtypes.

## Results

The 1457 cases of invasive breast cancer diagnosed during follow up included 998 (68%) HR-positive/HER2-negative, 120 (8%) HR-positive/HER2-positive, 44 (3%) HR-negative/HER2-positive, 138 (9%) HR-negative/HER2-negative breast cancers, and 157 (11%) with missing data on expression status for at least one receptor. The characteristics of cohort participants are shown in Table 1: the majority of participants (88%) were of non-Hispanic, white ethnicity and the median age at the time of the 10-year follow-up survey was 61 years (interquartile range 54–71 years). Participants had a relatively high prevalence of some of the established risk factors for breast cancer, with 20% reporting current use of HT and 15% reporting current consumption of ≥20 g of alcohol per day.

**Table 1:** Participant characteristics and use of non-steroidal anti-inflammatory medications, California Teachers Study, 2005–2012

Characteristic	Number	Percentage
Age (years)^a^		
<40	1781	3.1
40–49	7020	12.3
50–59	16,651	29.1
60–69	15,779	27.6
70–79	10,482	18.3
80–89	4812	8.4
≥ 90	639	1.1
Race^b^		
White	50,250	87.9
Hispanic	2220	3.9
Asian/Pacific Islander	2135	3.7
Black	1183	2.1
Other/mixed	972	1.7
Missing	404	0.7
Age at menarche (years)^b^		
<12	12,799	22.4
≥ 12	43,635	76.3
Missing	730	1.3
Age at first full-term pregnancy (years)^b^		
Nulliparous	14,157	24.8
< 25	15,171	26.5
≥ 25	26,909	47.1
Missing	927	1.6
Total time breastfeeding (months)^b^		
Never or <12	40,567	71.0
≥ 12	15,446	27.0
Missing	1,151	2.0
History of a benign breast biopsy^b^		
No	47,812	83.6
Yes	9175	16.1
Missing	177	0.3
Family history of breast cancer (mother or sister)^b^		
No	48,736	85.3
Yes	6763	11.8
Missing data or participant was adopted	1665	2.9
History of hospitalization for myocardial infarction^c^		
No	56,586	99.0
Yes	578	1.0
History of diabetes mellitus^a^		
No	54,433	95.2
Yes	2715	4.8
Missing	16	0.0
Strenuous plus moderate physical activity in the past 3 years (hours per week)^a^		
<3	26,079	45.6
≥3	30,909	54.1
Missing	176	0.3
Alcohol consumption (grams per day)^a^		
None or <20	46,033	80.5
≥20	8652	15.1
Missing	2479	4.3
Body mass index (kg/m^2^)^a^		
<25.0	29,509	51.6
25.0–29.9	16,619	29.1
≥30.0	10,359	18.1
Missing	677	1.2
Menopausal status and hormone therapy (HT) use^a^		
Premenopausal	6314	11.1
Perimenopausal or postmenopausal		
No HT in the last 5 years	27,397	47.9
Used HT in the last 5 years, but not currently using	10,142	17.7
Current HT use	11,420	20.0
Unknown menopausal status or HT use	1891	3.3
Regular-dose aspirin use at baseline^b^		
No NSAID use^d^	37,075	64.9
No regular-dose aspirin use, but used ibuprofen	7002	12.3
Used 1–3 days/week	6340	11.1
Used 4+ days/week	5732	10.0
Unknown	1015	1.8
Low-dose aspirin use at the 10-year follow up^a^		
Never in the past 3 years		
No NSAID use in the past 3 years^e^	21,421	37.5
No low-dose aspirin use, use of 1+ type(s)	15,678	27.4
No low-dose aspirin use, unknown use of 1+ type(s)	1829	3.2
Former	2766	4.8
Current, 1–2 tablets/week	915	1.6
Current, 3–4 tablets/week	1163	2.0
Current, 5–6 tablets/week	1406	2.5
Current, 7+ tablets/week	10,421	18.2
Unknown	1565	2.7
Regular-dose aspirin use at the 10-year follow up^a^		
Never in the past 3 years		
No NSAID use in the past 3 years^e^	21,421	37.5
No regular-dose aspirin use, use of 1+ type(s)	23,451	41.0
No regular-dose aspirin use, unknown use for 1+ type(s)	2253	3.9
Former/irregular	2378	4.2
Current, 3+ tablets/week	6387	11.2
Unknown	1274	2.2
Ibuprofen use at the 10-year follow up^a^		
Never in the past 3 years		
No NSAID use in the past 3 years^e^	21,421	37.5
No ibuprofen use, use of 1+ type(s)	18,219	31.9
No ibuprofen use, unknown use for 1+ type(s)	1499	2.6
Former/irregular	3679	6.4
Current, 3+ tablets/week	10,382	18.2
Unknown	1964	3.4
Other non-steroidal use at the 10-year follow up^a^		
Never in the past 3 years		
No NSAID use in the past 3 years^e^	21,421	37.5
No other non-steroidal use, use of 1+ type(s)	25,529	44.7
No other non-steroidal use, unknown use for 1+ type(s)	2307	4.0
Former/irregular	2301	4.0
Current, 3+ tablets/week	4263	7.5
Unknown	1343	2.4
Cox-2 inhibitor use at the 10-year follow up^a^		
Never in the past 3 years^e^		
No NSAID use in the past 3 years^e^	21,421	37.5
No Cox-2 inhibitor use, use of 1+ type(s)	28,055	49.1
No Cox-2 inhibitor use, unknown use for 1+ type(s)	2625	4.6
Former/irregular	2807	4.9
Current, 3+ tablets/week	1064	1.9
Unknown	1192	2.1

^a^From the 10-year follow-up questionnaire completed in 2005–2006. ^b^From the baseline questionnaire completed in 1995–1996. ^c^From linkage with the Office of Statewide Health Planning and Development (OSHPD) hospital discharge database from 1991 until the 10-year follow-up questionnaire. ^d^Included aspirin and ibuprofen used regularly (at least once a week) reported on the baseline questionnaire. ^e^Included aspirin, low-dose aspirin, ibuprofen, Cox-2 inhibitors, and other NSAIDs currently used regularly (at least once a week) reported on the 10-year follow-up questionnaire. *NSAID* non-steroidal anti-inflammatory drug

The most common NSAID reported as currently used by participants at the 10-year follow-up was low-dose aspirin (Table 1); 23% of women reported current use of at least three low-dose aspirin tablets per week. Ibuprofen was the second most common pain-relieving medication used, with 18% of participants reporting use of at least three tablets per week. Use of at least three regular-dose (325 mg) aspirin tablets per week was reported by 11% of the CTS participants. Less than 10% of participants reported current use of other NSAIDs or COX-2 inhibitors.

Current use of at least three tablets per week of any NSAID was not statistically significantly associated with any particular subtype of breast cancer or breast cancer overall (Table 2). However, when low-dose aspirin was considered separately, current use of three or more tablets per week of low-dose aspirin was statistically significantly associated with risk of breast cancer overall (HRR = 0.84, 95% CI 0.72–0.98 compared to those not taking any NSAIDS); this association was observed only for the HR-positive/HER2-negative subtype (HRR = 0.80, 95% CI 0.66–0.96). Considering more granular categories of frequency of low-dose aspirin use, relative to women who had not used any NSAID in the past 3 years, an inverse association with breast cancer risk was observed among women using 3–6 tablets per week (HRR = 0.72, 95% CI 0.54–0.96) and a marginal association was observed among those using 7+ tablets per week (HRR = 0.87, 95% CI 0.74–1.02). A similar pattern of inverse association was observed for the HR-positive/HER2-negative breast cancer subtype (for 3–6 tablets/week, HRR = 0.66, 95% CI 0.47–0.94; for 7+ tablets/week, HRR = 0.83, 95% CI 0.68–1.01, data not shown). We also observed a lower risk of breast cancer among women who reported current use of “other” NSAIDs (HRR = 0.79, 95% CI 0.62–1.00). Risk of HR-positive/HER2-positive tumors was increased in those with unknown use of ibuprofen, Cox-2 inhibitors, and “other” NSAIDs, but was based on a small number of cases (*n* = 13). Significant associations did not appear to differ by overweight status (*p* interaction = 0.18 for low-dose aspirin and all subtypes of breast cancer.)

**Table 2:** NSAID use and risk of breast cancer overall and by subtype, California Teachers Study, 2005–2012

		Breast cancer^a^	HR+/HER2–^a^	HR+/HER2+^b^	HR–/HER2−^c^	HR–/HER2+^d^
Any NSAID^e^						
No NSAID past 3 years	Cases, *n*	514	349	35	55	17
	HRR	1.0	1.0	1.0	1.0	1.0
Former/irregular	Cases, *n*	154	105	11	12	5
	HRR	1.04	1.04	1.11	0.79	1.03
	95% CI	0.86–1.24	0.83–1.29	0.56–2.20	0.42–1.48	0.38–2.81
Current, 3+ tablets/week	Cases, *n*	692	481	65	58	18
	HRR	0.90	0.91	1.30	0.77	0.74
	95% CI	0.80–1.01	0.79–1.05	0.86–1.98	0.53–1.12	0.38–1.45
Unknown	Cases, *n*	97	63	9	13	4
	HRR	1.16	1.09	1.73	1.58	1.45
	95% CI	0.93–1.44	0.83–1.43	0.83–3.61	0.86–2.90	0.48–4.37
Low-dose aspirin						
No NSAID past 3 years^e^	Cases, *n*	514	349	35	55	17
	HRR	1.0	1.0	1.0	1.0	1.0
No low-dose aspirin use but use of 1+ type(s)	Cases, *n*	400	281	34	33	10
	HRR	0.99	0.93	1.21	1.19	2.59
	95% CI	0.82–1.19	0.75–1.17	0.64–2.29	0.63–2.25	0.75–8.94
Former/irregular	Cases, *n*	104	71	7	6	4
	HRR	0.95	0.88	1.06	0.70	1.55
	95% CI	0.75–1.19	0.67–1.16	0.45–2.50	0.29–1.67	0.48–4.99
Current, 3+ tablets/week	Cases, *n*	338	230	33	31	10
	HRR	0.84	0.80	1.37	0.96	1.06
	95% CI	0.72–0.98	0.66–0.96	0.81–2.32	0.59–1.55	0.46–2.45
Unknown	Cases, *n*	101	67	11	13	3
	HRR	0.84	0.73	2.12	1.61	0.93
	95% CI	0.62–1.14	0.50–1.07	0.84–5.34	0.68–3.79	0.19–4.68
Regular-dose aspirin						
No NSAID past 3 years^e^	Cases, *n*	514	349	35	55	17
	HRR	1.0	1.0	1.0	1.0	1.0
No regular-dose aspirin use but use of 1+ type(s)	Cases, *n*	587	395	53	51	19
	HRR	0.96	0.90	1.35	1.19	2.10
	95% CI	0.80–1.16	0.72–1.12	0.72–2.51	0.64–2.22	0.65–6.82
Former/irregular	Cases, *n*	73	59	4	4	1
	HRR	1.12	1.27	1.03	1.03	0.96
	95% CI	0.84–1.50	0.91–1.77	0.32–3.31	0.34–3.13	0.11–8.30
Current, 3+ tablets/week	Cases, *n*	170	119	18	14	3
	HRR	0.97	0.95	1.68	1.02	0.84
	95% CI	0.80–1.18	0.75–1.20	0.90–3.17	0.54–1.93	0.24–3.03
Unknown	Cases, *n*	113	76	10	14	4
	HRR	1.13	1.04	1.70	2.21	2.35
	95% CI	0.83–1.54	0.72–1.52	0.60–4.81	0.91–5.37	0.29–18.94
Ibuprofen						
No NSAID past 3 years^e^	Cases, *n*	514	349	35	55	17
	HRR	1.0	1.0	1.0	1.0	1.0
No ibuprofen use but use of 1+ type(s)	Cases, *n*	472	317	43	44	19
	HRR	0.96	0.92	1.14	1.19	2.22
	95% CI	0.80–1.15	0.74–1.15	0.61–2.14	0.64–2.23	0.69–7.16
Former/irregular	Cases, *n*	93	68	9	5	0
	HRR	0.95	1.00	1.29	0.65	
	95% CI	0.74–1.23	0.74–1.35	0.56–2.97	0.24–1.76	
Current, 3+ tablets/week	Cases, *n*	263	190	20	20	4
	HRR	1.04	1.09	1.00	0.90	0.66
	95% CI	0.88–1.23	0.90–1.34	0.54–1.84	0.51–1.58	0.21–2.10
Unknown	Cases, *n*	115	74	13	14	4
	HRR	1.30	1.12	2.59	1.97	1.18
	95% CI	0.99–1.70	0.79–1.56	1.16–5.79	0.90–4.31	0.19–7.46
Other non-steroidal						
No NSAID past 3 years^e^	Cases, *n*	514	349	35	55	17
	HRR	1.0	1.0	1.0	1.0	1.0
No other non-steroidal use but use of 1+ type(s)	Cases, *n*	662	456	57	59	20
	HRR	0.96	0.91	1.21	1.21	2.05
	95% CI	0.80–1.15	0.73–1.13	0.65–2.25	0.65–2.26	0.63–6.63
Former/irregular	Cases, *n*	64	38	7	6	3
	HRR	0.98	0.77	1.62	1.41	3.14
	95% CI	0.72–1.33	0.52–1.13	0.62–4.20	0.54–3.64	0.78–12.72
Current, 3+ tablets/week	Cases, *n*	96	75	8	4	0
	HRR	0.79	0.85	0.97	0.43	
	95% CI	0.62–1.00	0.64–1.13	0.41–2.25	0.15–1.26	
Unknown	Cases, *n*	121	80	13	14	4
	HRR	1.17	1.00	2.74	1.91	1.94
	95% CI	0.86–1.58	0.69–1.45	1.09–6.87	0.79–4.62	0.38–9.87
Cox-2 inhibitor						
No NSAID past 3 years^e^	Cases, *n*	514	349	35	55	17
	HRR	1.0	1.0	1.0	1.0	1.0
No Cox-2 inhibitor use, but use of 1+ type(s)	Cases, *n*	706	492	59	59	19
	HRR	0.96	0.90	1.22	1.22	2.68
	95% CI	0.80–1.15	0.72–1.12	0.66–2.27	0.66–2.28	0.78–9.17
Former/irregular	Cases, *n*	82	57	8	6	4
	HRR	1.02	0.97	1.64	1.16	3.81
	95% CI	0.78–1.34	0.70–1.34	0.68–3.97	0.46–2.96	1.07–13.57
Current, 3+ tablets/week	Cases, *n*	30	15	5	4	1
	HRR	0.92	0.63	2.60	1.74	1.74
	95% CI	0.63–1.34	0.37–1.07	0.97–7.00	0.61–4.99	0.22–13.66
Unknown	Cases, *n*	125	85	13	14	3
	HRR	1.19	1.09	3.08	1.94	0.88
	95% CI	0.88–1.61	0.75–1.58	1.21–7.88	0.79–4.75	0.15–5.22

^a^Cox regression models used age as the time metric, were stratified by age at the follow-up questionnaire, and were adjusted for age at menarche, parity and age at first full-term pregnancy, total months breastfeeding their offspring, history of a benign breast biopsy, family history of breast cancer (﻿mother or sister), strenuous plus moderate physical activity, alcohol consumption, body mass index, menopausal status and hormone therapy use, and (except for “Any NSAID”) all of the other NSAIDS in the table (for each type: never past 3 years, former/irregular, current 3+ tablets/week, unknown). ^b^Cox regression models used age as the time metric, were stratified by age at the follow-up questionnaire, and were adjusted for parity and age at first full-term pregnancy, body mass index, and (except for “Any NSAID”) all of the other NSAIDS in the table. ^c^Cox regression models used age as the time metric, were stratified by age at the follow-up questionnaire, and were adjusted for race, alcohol consumption and its interaction with time-dependent age, and (except for “Any NSAID”) all of the other NSAIDS in the table. ^d^Cox regression models used age as the time metric, were stratified by age at the follow-up questionnaire, and were adjusted for total months breastfeeding their offspring, history of a benign breast biopsy, and (except for “Any NSAID”) all of the other NSAIDS in the table. ^e^Included aspirin, low-dose aspirin, ibuprofen, Cox-2 inhibitor, and other NSAIDs currently used regularly (at least once a week) reported on the 10-year follow-up questionnaire. *NSAID* non-steroidal anti-inflammatory drug, *HRR* hazard rate ratio, *CI* confidence interval

To assess associations with risk of breast cancer ﻿overall and with risk of one subtype among probable long-term users of NSAIDs, we carried out secondary analyses combining information reported on the baseline and 10-year follow-up questionnaires. Among women currently using 3+ low-dose aspirin tablets per week at the 10-year follow up, risk of breast cancer was comparable between those who reported no NSAID use at baseline (HRR = 0.79, 95% CI 0.66–0.95, 181 cases) and those who reported using aspirin regularly for 4+ days per week at baseline (HRR = 0.79, 95% CI 0.59–1.08, 50 cases) compared to those who reported no NSAID use on both the baseline questionnaire and the 10-year follow-up questionnaire (409 cases). Results were similar for those currently using 3+ tablets per week of regular-dose aspirin at the 10-year follow-up (HRR = 0.92, 95% CI 0.70–1.20 (67 cases) for no NSAID use at baseline; HRR = 0.94, 95% CI 0.67–1.31 (39 cases) for aspirin 4+ days per week at baseline). Similar patterns were observed for the HR-positive/HER2-negative subtype (data not shown).

To assess possible confounding by indication, we also assessed associations between breast cancer and acetaminophen, a pain reliever that is not an NSAID. Current use of at least 3 tablets per week of acetaminophen was not associated with breast cancer or any particular subtype of breast cancer in models adjusted for the five NSAIDS (for breast cancer, HRR = 1.00, 95% CI 0.87–1.15 for current use of 3+ tablets per week of acetaminophen (264 cases) compared to no use of acetaminophen in the last 3 years (1109 cases); for the HR-positive/HER2-negative subtype, HRR = 0.98, 95% CI 0.83–1.16 for current use of 3+ tablets per week (180 cases) compared to no use of acetaminophen in the last 3 years (757 cases); other data not shown).

## Discussion

Among CTS participants who reported their detailed, current use of NSAIDs and then were followed prospectively for a median of 7 years, the association between use of specific NSAIDs and the development of invasive breast cancer or its receptor-defined subtypes differed depending on the NSAID used. In the 23% of women who reported using low-dose aspirin at least three times per week, we observed a modest 20% reduction in risk of developing HR-positive/HER2-negative breast cancer, which is likely responsible for the similar association observed between NSAID use and risk of breast cancer overall. This association persisted after consideration of other breast cancer risk factors including use of HT and prior history of myocardial infarction. This association is intriguing because no such association was observed with the use of regular-dose aspirin (325 mg). We suspect that this could relate to the more regular use of low-dose aspirin for cardioprotection, as opposed to a more sporadic pattern of use of regular-dose aspirin to relieve pain. We did not observe any apparent modification of the low-dose aspirin effect by overweight status, nor did we observe stronger associations in women who were likely to be longer-term users, having reported using aspirin 10 years earlier, at baseline. This association should be re-examined in cohorts with larger numbers of incident breast cancers in which HR and HER2 status are recorded.

The three studies published previously that assessed HR-defined and HER-2-defined subtypes differed in the definitions of medication dose and duration of use, limiting our ability to compare the results of those studies to ours. The Nurses’ Health Study [10] conducted the most comprehensive assessment of NSAID use in relation to receptor-defined subtypes, finding no association between breast cancer and use of non-aspirin NSAIDs or acetaminophen. They defined regular users of aspirin as those using two or more reduced tablets per week, but did not distinguish low-dose from regular-dose formulations. Across the four HR/HER2-defined subtypes, the analyses of which were based on fewer cases than in the present study, statistically significant protective effects against HR-positive/HER2-negative breast cancer were detected (n = 341 cases), regardless of the duration of reported use (10+ years of use: relative risk (RR) = 0.66, 95% CI 0.49–0.89, fewer than 10 years of use: RR = 0.75, 95% CI 0.58–0.96). They did not observe any association with the HR-positive/HER2-positive subtype (n = 74 cases, 10+ years of use: RR = 1.47, 95% CI 0.76–2.82; fewer than 10 years of use: RR = 1.40, 95% CI 0.79–2.51) or for the receptor-negative subtypes (n = 174 cases), or for breast cancer overall [16, 17]. This pattern of association is similar to what we have shown here for CTS participants.

In the Nashville Breast Health Study [12], a case-control study, the authors reported statistically significant protective effects of regular use of any NSAID against the risk of all receptor-defined subtypes, with a reduced odds ratio (OR) for HR-positive/HER2-negative cancer (OR = 0.71, 95% CI 0.56–0.88) similar to the HRR reported here; this was limited to overweight women with BMI of at least 25 kg/m^2^ [12]. Our results did not suggest an interaction with overweight status. In the Western New York Exposures and Breast Cancer Study [11], another case-control study, the authors reported a statistically significant reduction in risk of breast cancer overall associated with aspirin use, which did not persist for any subtype examined, including the four receptor-defined subtypes.

Our key finding is related to low-dose aspirin and not to regular-dose aspirin. Women who reported using low-dose aspirin were more likely to take it more than three times per week or daily, possibly for cardiovascular disease prevention. In our prior analysis of NSAID use reported at baseline by CTS participants and development of subsequent breast cancer [18], we could not distinguish between low-dose and regular-dose aspirin as we did not inquire about dose in the baseline questionnaire. In that analysis we found no association between use of aspirin or ibuprofen more than once weekly and risk of breast cancer overall, but risk of HR-negative breast cancer was increased with 5 or more years of daily aspirin use (RR = 1.81, 95% CI 1.12–2.92) [18]. In the current assessment, we did not see any association between breast cancer and use of regular-dose aspirin, nor did we detect any significant increase in risk of any breast cancer subtype with any aspirin use, regardless of dose. Our assessment of probable long-term users (women who used aspirin at baseline and at the 10-year follow-up) showed that these women had a similar risk of breast cancer overall and of the HR-positive/HER2-negative subtype as those who reported current use at the 10-year follow-up.

Our finding of an inverse association between use of 3+ low-dose aspirin tablets/week and risk of breast cancer overall is consistent with the findings of several other observational studies that did not separately examine HR-defined and HER2-defined subtypes. The largest and most detailed prospective study, the VITAL cohort [19], found that women taking 81 mg low-dose aspirin for ≥4 days per week had a more pronounced reduction in risk of breast cancer overall (HRR = 0.65, 95% CI 0.43–0.97) after 10 years of follow up [20] than we observed here. The results from a large prospective study using the UK General Practice Database [21] showed a statistically significant decreased risk of breast cancer among women who took low-dose aspirin daily for at least 1 year (OR = 0.67, 95% CI 0.51–0.89), suggesting only a short duration of use was need﻿ed for a reduction in risk to become apparent.

In contrast to these studies, in the Women’s Health Initiative observational study [22] risk of breast cancer overall was reduced 21% in women who took regular-dose aspirin but not in women who took low-dose aspirin. Our findings are not consistent with the Women’s Health Study, a randomized clinical trial of the use of low-dose (100 mg) aspirin every other day, a frequency that would have mapped to the low end of our category defining regular use as at least three tablets weekly. After 10 years, the risk of breast cancer overall was unchanged (RR = 0.98, 95% CI 0.97–1.09, *p* = 0.68) [3] among the women randomized to low-dose aspirin. In a later sub-analysis the investigators reported no association according to breast tumor characteristics such as size, histology, grade, or differentiation [17]. Altogether, these studies support the notion that use of low-dose aspirin at least three times per week, or perhaps daily, modestly reduces overall breast cancer risk by about 20–25%. Our results add to this evidence base, suggesting that the reduction in risk occurs mainly in the HR-positive/HER2-negative subtype.

The biological mechanism by which low-dose aspirin could function as a chemopreventive agent against HR-positive/HER2-negative breast cancer, but not other breast cancer subtypes, is not yet clear. A consistent lowering of COX-2 and prostaglandin activity could prevent or slow carcinogenesis in a number of ways, at the tumor level, by interfering with DNA adduct formation [23], inhibiting tumor angiogenesis [4, 24, 25], or promoting apoptosis [26]. Recent data examining serum circulating inflammatory markers among healthy subjects ages 55–74 years did not indicate that regular-dose aspirin use is associated with any of 78 circulating markers, calling into question the relevance of circulating levels of immune markers [27] and raising the possibility of a more local immune effect. Alternatively, as prostaglandins may upregulate production of circulating estrogens via aromatase [28, 29], daily use of low-dose aspirin may inhibit aromatase, which could reduce levels of key hormones and thereby impact initiation or promotion of estrogen-sensitive tumors [18, 30, 31]. This analysis and that from the Nurses’ Health Study [10] indicated protective effects of consistent aspirin use against the risk of HR-positive tumors, but only those that are also HER2-negative. On the other hand, elevated COX-2 levels have been detected in triple-negative tumors [32].

Strengths of this study include the complete and accurate prospective ascertainment of HER2-defined breast cancer development based on routine linkage of the cohort to the statewide cancer registry, linkage to hospital discharge summary data to confirm any previous myocardial infarction, and a median of 7 years of follow up. Our study also had limitations. The total number of breast cancer cases was greater than was available in previously published analyses, but t﻿he ﻿lim﻿ited ﻿﻿numbers available for subtype-specific analyses meant that these﻿ were exploratory. It is possible that women who regularly take low-dose aspirin differed from women who did not, based on important health parameters (other than history of myocardial infarction or comorbid diabetes mellitus), resulting in residual and unmeasured confounding. For instance, women who regularly take low-dose aspirin could engage in more health-conscious behavior than non-users or infrequent-users. Like most observational studies, we were only able to measure NSAID exposure as “snapshots” of exposure at the time women completed their surveys. We were limited in evaluating possible confounding by indication for cardiovascular disease that did not result in hospitalization for myocardial infarction. Finally, this population is not representative of the general population of California women or women across the USA, particularly with respect to educational status; thus, it is uncertain how generalizable our findings are to the broader population, particularly non-white women and women born outside the USA. At cohort inception in 1996, CTS participants had incidence rates of breast cancer that were over 50% higher than those for age-matched and race-matched women in California [14], which probably reflects higher prevalence of risk factors including hormone therapy use, alcohol consumption and particular reproductive profiles.

In summary, our study strongly supports the need for further, perhaps experimental, study of low-dose aspirin as a widely available, inexpensive chemopreventive option for the most common subtype of breast cancer, the HR-positive/HER2-negative subtype. Our study adds to the existing evidence on this topic, showing that previously reported associations between low-dose aspirin use and risk of breast cancer overall may be driven by a more specific association with this hormone-sensitive and HER2-negative breast cancer subtype. Furthermore, it suggests that previously reported associations using measures that combined low-dose ﻿aspir﻿in us﻿e﻿ (more likely to be daily or more frequent) with regular aspirin use should reassess these associations. Future studies of aspirin and breast cancer must be able to distinguish low-dose from other formulations and to assess risks separately by molecularly defined subtype. Such studies should not only detail this chemopreventive potential but should also quantify any side effects associated with regular low-dose aspirin use.

## Conclusions

For 23% of women who reported using low-dose aspirin at least three times per week, we observed a modest 20% reduction in risk of developing HR-positive/HER2-negative breast cancer, which is likely responsible for the similar association observed for breast cancer overall. No such association was observed for use of regular-dose aspirin (325 mg) or other NSAIDs. We suspect that our observations could relate to the pattern of daily use of low-dose aspirin for prevention, as opposed to more sporadic patterns of use to relieve pain. Our data are intriguing as regards the role of low-dose aspirin in breast cancer prevention but this question should be revisited in cohorts with larger numbers of incident breast cancers, in which HR and HER2 status are also recorded.
